# Tracing and testing multiple generations of contacts to COVID-19 cases: cost–benefit trade-offs

**DOI:** 10.1098/rsos.211927

**Published:** 2022-10-12

**Authors:** Jungyeol Kim, Xingran Chen, Hesam Nikpey, Harvey Rubin, Shirin Saeedi Bidokhti, Saswati Sarkar

**Affiliations:** ^1^ Department of Electrical and Systems Engineering, University of Pennsylvania, Philadelphia, PA 19104, USA; ^2^ Department of Computer and Information Science, University of Pennsylvania, Philadelphia, PA 19104, USA; ^3^ Department of Medicine, University of Pennsylvania, Philadelphia, PA 19104, USA

**Keywords:** disease dynamics, contact tracing, cost–benefit trade-off, COVID-19, large-scale contact networks

## Abstract

Traditional contact tracing tests the direct contacts of those who test positive. But, by the time an infected individual is tested, the infection starting from the person may have infected a chain of individuals. Hence, why should the testing stop at direct contacts, and not test secondary, tertiary contacts or even contacts further down? One deterrent in testing long chains of individuals right away may be that it substantially increases the testing load, or does it? We investigate the costs and benefits of such multi-hop contact tracing for different number of hops. Considering diverse contact networks, we show that the cost–benefit trade-off can be characterized in terms of a single measurable attribute, the *initial epidemic growth rate*. Once this growth rate crosses a threshold, multi-hop contact tracing substantially reduces the outbreak size compared with traditional tracing. Multi-hop even incurs a lower cost compared with the traditional tracing for a large range of values of the growth rate. The cost–benefit trade-offs can be classified into three phases depending on the value of the growth rate. The need for choosing a larger number of hops becomes greater as the growth rate increases or the environment becomes less conducive toward containing the disease.

## 1. Introduction

To slow down the spread of COVID-19, public health authorities like the US Center for Disease Control and Prevention (CDC) recommended testing those who have in the recent past been in physical proximity with an individual who has tested positive, even when the contacts do not exhibit any symptom [1]. This pre-emptive action, commonly known as contact tracing, is deployed because, given how contagious the disease is, a patient is likely to have passed the virus to their contacts, and the infected contacts have the potential to infect others even before they show symptoms [2]. Discovering and quarantining those infected contacts will stop them from spreading the disease much earlier than a strategy in which only symptomatic individuals who seek medical help are tested. Slowing down the spread by contact tracing comes at the cost of an increase in the testing load, yet, the cost–benefit trade-off for contact tracing is understood to be substantially favourable, as compared with universal lockdowns, for example, which has led to economic downturns in several countries.

In this paper, we want to understand under what circumstances traditional contact tracing alone is sufficient to contain the virus and why such containment is attainable in those circumstances. We also want to understand circumstances where the traditional approach is not efficient enough and how we can overcome this. A question that naturally arises in this regard is if cost–benefit trade-offs may be enhanced through natural generalizations of the core concept of contact tracing—this is what we seek to answer in this paper. In the time that elapses between when an individual, *i*, is infected until *i* is tested, the disease spreads from *i* through a chain of several hops—*i* infects those *i* is in contact with, those whom *i* infects can infect their contacts, the infected contacts can infect their contacts, and so on. A recent study suggests that, due to the high speed of transmission, the epidemic may continue to grow even if all contacts are quarantined with some delay [3].

**Figure 1.**
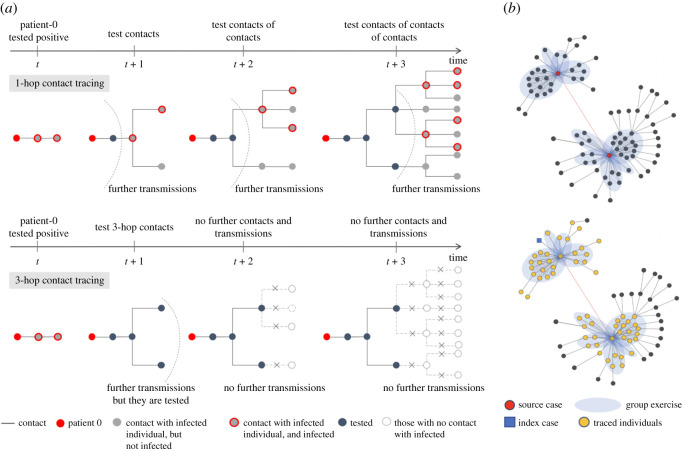
(*a*) We use an example to illustrate and compare 1-hop contact tracing shown on top (i.e. tracing and testing only the direct contacts of those who test positive) and 3-hop contact tracing shown on bottom (i.e. tracing and testing the direct, secondary and tertiary contacts of those who test positive). Here, at time *t* when patient-0 (red) is tested by a health authority, the infection has already propagated 2 hops. By time *t* + 3, both tracing policies test four individuals (marked in black) other than the patient-0; the 3-hop policy tests and quarantines the positive ones in a shorter time, while 1-hop tests and quarantines them progressively and therefore over longer times. Accordingly, only three individuals are infected under the 3-hop policy, while 10 individuals are infected under the 1-hop policy. (*b*) Top network: this figure is a partial network based on epidemiological investigation information by the Korea Centers for Disease Control and Prevention (KCDC) and local governments [4]. It illustrates how infection spreads from two dance instructors (source cases; red circle), both of whom attended a workshop on 15 February 2020, in South Korea. Subsequently, they separately taught dance classes indoor and spread to the attendees who spread to their contacts. The blue shaded area represents the instructors and the attendees in each dance class, and the close contacts (grey dashed line edges) among them in the class. The grey solid lines represent the contact during which the disease is transmitted. The dashed red line between the two instructors indicate that they were in contact (because they simultaneously attended the workshop). Bottom network: Suppose an index case indicated by the blue square is identified by the health authority. When multi-hop (e.g. 3 hop) contact tracing is done, the traced nodes also form a cluster. Thus, intuitively, the growth of the traced cluster emulates the growth of the infected cluster; this emulation helps in containment.

Fewer people are likely to be infected by testing and quarantining not only direct contacts of an individual who tests positive, but contacts of the contacts and so on (figure 1*a*). Such *multi-hop* tracing and testing will enable identification and quarantine of the individuals further down the chain who have been exposed, earlier than if we had tested only the direct contacts of those who have tested positive and then reach down the chain iteratively. To see why multi-hop contact tracing may be effective, note that an infectious disease spreads through growth of clusters of infected individuals around one or more origins, e.g. during the spread of COVID-19, large clusters were observed in meat-packing plants in seven countries, and an e-commerce distribution warehouse in South Korea [5]. Contact tracing also forms clusters of tested individuals that grow from and around one or more individuals who initially test positive (figure 1*b*). In this sense, contact tracing emulates the spread of the disease. If the testing cluster grows faster than the infection cluster and also substantially overlaps with the latter, the outbreak will be contained. And by virtue of its design, multi-hop contact tracing grows the testing cluster faster than traditional contact tracing.

Multi-hop tracing strategies have been sporadically deployed in practice with considerable success. For example, in Vietnam, public health authority sometimes reached out to tertiary contacts, and found and tested as many as 200 contacts for each case [6]; many of those who were traced and quarantined during the first 100 days of the pandemic were in fact secondary contacts of those who tested positive [7]. Vietnam reported only a total of 1465 PCR-confirmed cases and 35 deaths by the end of 2020 [8]. Nonetheless, this concept has not been comprehensively and systemically investigated—this is the void this paper seeks to fill in.

The following questions arise in context of multi-hop tracing: (i) Under what circumstances can traditional contact tracing not significantly reduce the outbreak size? In these cases, do aggressive pre-emptive tracing schemes under multi-hop reduce the outbreak size significantly? (ii) Do such schemes necessarily increase the overall number of tests and quarantines? The answer is not *a priori* clear as reduction in overall infection spread through such a strategy may eventually reduce the number of tests required, as illustrated in figure 1*a*. (iii) If multi-hop tracing turns out to be beneficial, how many hops provide the best cost–benefit trade-off? Does a *saturation phenomenon* in which the benefit increases only marginally by increasing the number of hops beyond a certain point arise? If so, what is the saturation point? (iv) How do these answers depend on the attributes of the tests, the time at which contact tracing is deployed, and the behavioural dynamics, that is, the extent of public compliance to public health directives? We proceed to answer these questions in this paper through stochastic simulation on diverse large-scale contact networks, spanning data-driven (i.e. real-world) networks, as also networks of a classical synthetic variety.

We evaluate the contact tracing and testing strategies through stochastic simulation which relies on a compartmental model for the spread of the disease. Compartmental models have been widely used in studies on virus spread [9,10]. Stochastic simulations have also been extensively used for evaluating various infectious disease control mechanism, e.g. testing [9], vaccination [11], use of masks [12], etc. Analytical models such as mean-field approximation strategies constitute complementary tools for studying the spread of infectious diseases and evaluating the efficacy of various disease control strategies. Classical mean-field approximations suffer from the limitation that the neighbour of every node is defined as a ‘socially averaged hypothetical neighbour’ which does not capture the heterogeneity of a real-world complex social network (as noted in [13]). Mean-field approximation strategies have been refined in various directions, e.g. pair approximation [13], degree-based approximation [14,15], metapopulation approximation [16], etc. (refer to [17] for further details). All these resort to some form of averaging of neighbourhoods or more generally groups of nodes, which do not again capture the local variations in a real-world complex social network. The results predicted by these approximations therefore exhibit discrepancies from the results predicted by stochastic simulation, the extent of the discrepancy depends on the approximation technique, the order of statistics considered, and network in question. Testing strategies, in particular contact tracing and its generalizations, often determine which nodes need to be tested depending on the number and identity of nodes that have been infected in neighbourhoods of certain sizes around each node. Thus, details of the networks are crucial in evaluating these. Thus, these are best evaluated based on stochastic simulation on contact networks in which nodes are individuals and edges are interactions between them.

We formalize the aggressive pre-emptive tracing and testing scheme as *k*-hop contact tracing, where *k* is the depth of the contact chain that is traced. For example, *k* = 0 does not trace contacts and tests only those who show symptoms and seek medical help, *k* = 1 is the traditional contact tracing that tests the direct contacts of an individual who tests positive, *k* = 2 additionally tests the contacts of the contacts, *k* = 3 tests yet another hop of contacts, and so on. We call the multiple generations of contacts to COVID-19 cases (i.e. *k*-hop contact tracing for *k* ≥ 2) *multi-hop contact tracing*.

We quantify the costs and benefit of contact tracing over a course of six months (180 days) starting from the day after contact tracing is initiated, and compare the results for multi-hop contact tracing with 1-hop contact tracing. The *benefit* is defined as the percentage of reduction in the number of infections over the period compared with when no contact tracing was performed. The *costs* comprise (i) the number of tests and (ii) total sum of days of quarantine for the entire population over the period.

However, the nature of the cost–benefit trade-off for multi-hop contact tracing depends on practical aspects of contact tracing and testing that are present in all types of infectious diseases. First of all, behavioural dynamics undermine the efficacy of contact tracing. Individuals do not always cooperate with public-health authorities by (i) disclosing their contacts and (ii) quarantining when exposed to those who test positive. Secondly, the tests suffer from false negatives and false positives. If an individual tests negative falsely, his *k*-hop contacts will not be traced and tested (unless those contacts are within *k*-hop of another individual who tests positive). This undermines the ability of the tracing strategy to contain the outbreak. False positives may increase cost by setting off a chain of unnecessary tracing and testing. Thirdly, tests can have different turnaround times, high turnaround times delay tracing the contacts of those infected. Lastly, contact tracing in its entirety may be initiated by public health officials only after the infection level in the target populace crosses a certain threshold. All these attributes are likely to affect the outcome of the tracing. These attributes depend on regional and cultural characteristics and public health policies which are different in different ambiences. Given the inherent uncertainty of the settings and the heterogeneity for different venues, we consider a range of values of the above attributes based on estimates available in the literature.

The dynamics of epidemic spread are governed by inter-personal contact patterns and probability with which a contagious individual infects a susceptible individual in an interaction. Thus, these factors uniquely determine *initial epidemic growth rate* that characterizes the intrinsic speed of virus spread within each community in the absence of any public health intervention. We consider diverse large-scale contact networks and a range of values of the transmission probability.

Under a variety of contact patterns and transmission probabilities, our simulations reveal that the nature of the cost–benefit trade-off for multi-hop contact tracing can be characterized in terms of the growth rate, and the nature remains largely stable to variation of the above-mentioned practical aspects in reasonable ranges. When the growth rate is low, 1-hop contact tracing alone can sufficiently contain the virus. However, once the growth rate crosses a threshold value, a sharp *phase transition* is exhibited. Specifically, at intermediate growth rates, the benefit that 1-hop provides dramatically decreases when compared with the low growth rate range, and multi-hop contact tracing offers substantial further benefit *even at a lower cost compared with 1-hop*. At high growth rates, multi-hop contact tracing provides substantial further benefit but incurs greater costs, when compared with 1-hop contact tracing.

Our results also reveal that the further benefit of adding more hops beyond 1-hop tends to diminish progressively in most settings, thus a *saturation phenomenon* is observed throughout. In general, the hop number at which the saturation phenomenon is observed becomes greater as the growth rate increases and/or the environments regarding practical aspects become less conducive toward containing the disease. Specifically, increasing the number of hops beyond 3-hop provides only marginal benefit in most cases, thus the saturation point is confined to 1, 2 and 3 hops, despite variations of all the above-mentioned attributes; the need for considering 4 and 5 hops largely arises for very limited conditions such as higher growth rates and more challenging environments.

## 2. Model dynamics and contact tracing process

We consider a discrete time stochastic evolution of COVID-19 on diverse large-scale contact networks, spanning a large number of networks of a classical synthetic variety and data-driven networks (see Data for details on networks). The disease spreads from the contagious individual (CI) to the susceptible individual (SI) through mutual interaction. In any given interaction with a CI, an SI is infected with a probability *β*. This transmission probability depends on a range of factors, such as whether the individuals observe social distancing and wear protective equipment, and varies from one venue to another. After a *latency period*, the newly infected individuals become contagious. Specifically, at the end of the latency period, the individuals either become *presymptomatic* (the stage before exhibiting symptoms), or *asymptomatic* (that is, they never show symptoms). Presymptomatics proceed to become *symptomatics* in the next stage. After a random delay, symptomatics opt for seeking medical help and testing, and become *ready-to-test*. Presymptomatics, asymptomatics and symptomatics all, however, are contagious. Refer to Methods for details on the systems we consider and the parameters we choose.

Once the individual in question tests positive, the public health authority traces his *k*-hop contacts, over the last 14 days, and informs them at the end of the day that they may have been exposed. Such contact tracing may be accomplished through digital contact tracing (refer to electronic supplementary materials for details on digital contact tracing pertaining to multi-hop contact tracing). The authority asks them to self-quarantine for 14 days unless they are already under quarantine or ever tested positive before. We assume that the traced individuals are scheduled for testing in 3 days. The test results are available in 1–3 days. The tested individuals continue the quarantine even if they test negative, because a negative test may be the result of a false negative. Now, those who tested negative can get traced and tested again if someone in their vicinity tests positive (because again the previous negative test may have been false negative).

## 3. Results

We quantify the costs and benefit of contact tracing over a course of six months (180 days) starting from the day after contact tracing is initiated. The total number of infections, the total number of tests and the total sum of days of quarantine for the entire population over the period are averaged over 150 simulation runs, excluding those in which fewer than 40 individuals are infected within the first three months (90 days). By comparing the mean values of these results for different number of hops, we evaluate cost–benefit trade-offs of multi-hop contact tracing scheme.

We consider an attribute called *initial epidemic growth rate*, or more simply the *growth rate*, that characterizes the intrinsic speed of virus spread within each community in the absence of any public health intervention. Using the data available in [18], we found that the growth rate for COVID-19 for different political units (142 country/region or province/state/dependency) range from 0 to 0.31, with a median of 0.12. The range of *β* that we consider provides initial epidemic growth rates, in the diverse contact networks we consider, in a range that subsumes the realistic range [0, 0.31]. Refer to Methods for the definition of growth rate and electronic supplementary materials for values of real-world growth rate.

*Cost–benefit metrics*. We first define key cost–benefit metrics that are used throughout the evaluations. Recall that the *benefit* is defined as the percentage of reduction in the number of infections over the period of study (a course of six months starting from the day after contact tracing is initiated) compared with when no contact tracing was performed. The benefit can be expressed as

3.1 $${\text{Benefit}}_{k\text{-hop}}=\frac{{\text{Total Infections}}_{k\text{-hop}}-{\text{Total Infections}}_{0\text{-hop}}}{{\text{Total Infections}}_{0\text{-hop}}}\times 100.$$

We next define *relative benefit* and *relative costs* to quantify the incremental benefits and costs multi-hop contact tracing provides/incurs as compared with 1-hop contact tracing. The *relative benefit for* *k*-*hop*, *k* > 1, is defined as the difference between the benefits provided by *k*-hop and 1-hop,

3.2 $${\text{Relative Benefit}}_{k\text{-hop}}={\text{Benefit}}_{k\text{-hop}}-{\text{Benefit}}_{1\text{-hop}}.$$

The *relative costs for k-hop*, *k* > 1, is defined as the ratio of cost difference between multi-hop and 1-hop to cost for 1-hop contact tracing,

3.3 $${\text{Relative Cost}}_{k\text{-hop}}=\frac{{\text{Cost}}_{k\text{-hop}}-{\text{Cost}}_{1\text{-hop}}}{{\text{Cost}}_{1\text{-hop}}}\times 100,$$

which represents how much more or less cost is required compared with 1-hop contact tracing. In this definition, the costs comprise quarantine cost and test cost. The *test cost* is defined as the total number of tests over the period of consideration; the *quarantine cost* is defined as the total sum of days of quarantine for the entire population over the period of consideration, which equals the number of days each individual is quarantined added over all individuals.

Note that the expressions for relative benefit and relative cost are structurally different in that the first is a difference while the second is a ratio. The reason for this structural disparity is that benefit is a relative value, while cost is an absolute value. So we compare (i) the benefits provided by *k*-hop and 1-hop through the difference between them and (ii) the costs incurred by *k*-hop and 1-hop through the ratio between them.

First, we study the benefits and costs for multi-hop contact tracing over single hop (i.e. traditional) contact tracing. We show that the cost–benefit trade-offs for multi-hop contact tracing can be classified into three phases, each corresponding to a different range of the growth rates; as the growth rate transitions into different ranges, sharp phase transitions are often observed. Subsequently, we compare the multi-hop contact tracing strategy with a complete lockdown (i.e. every individual is quarantined). The comparison shows that multi-hop contact tracing can effectively control the spread of the virus while eliminating a large volume of unessential quarantines which inevitably arise in a complete lockdown. Lastly, we reveal that the further benefit for adding another hop beyond 1-hop tends to diminish progressively, thus a saturation phenomenon is observed. Accordingly, we investigate the highest number of hops (saturation point) that can lead to a non-negligible further reduction in the number of infections. We show that the saturation point increases as the growth rate increases and the environment becomes less conducive toward containing the disease, and is confined to 1, 2 and 3 hops in most cases.

### 3.1. Single-hop versus multi-hop contact tracing: a cost–benefit perspective

#### 3.1.1. Synthetic networks

**Figure 2.**
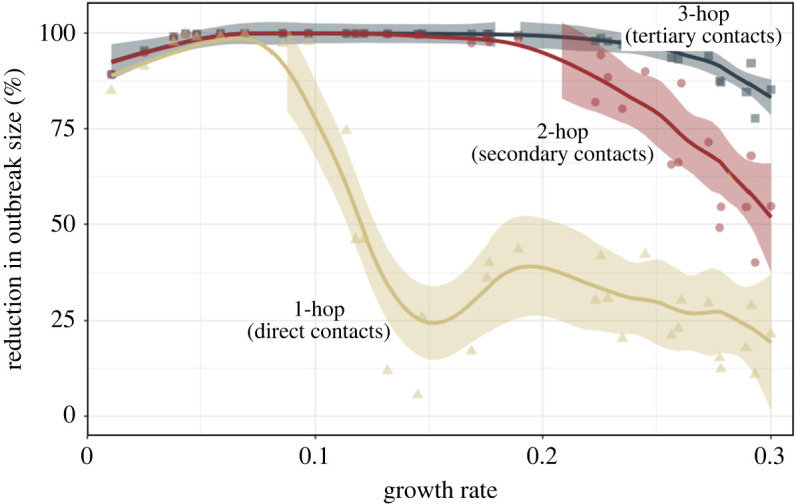
The percentage of reduction in the number of infections when 1-hop, 2-hop and 3-hop contact tracing policies were performed, compared with when no contact tracing was performed, as a function of the growth rate. Simulation results on different networks (both synthetic and data-driven networks) and parameter combinations constitute points on a plot with growth rate as the horizontal axis and benefit as the vertical axis. For example, a point where the *y*-axis corresponds to 20% indicates that the number of infections that would occur when no contact tracing is performed can be reduced by 20% through the implementation of the contact tracing policy. Tracing secondary (2-hop) and tertiary (3-hop) contacts can reduce the outbreak size significantly in cases of high growth rate where tracing direct (1-hop) contacts alone cannot sufficiently reduce the outbreak size. The solid lines correspond to the locally estimated scatterplot smoothing (LOESS) smoother with a span value of 0.3 and the shadings represent the 95% confidence interval around the smoother line.

We first evaluate the cost–benefit trade-offs of multi-hop contact tracing under the default scenario for the synthetic networks. Figure 2 represents different networks and parameter combinations as points on a plot with growth rate as the horizontal axis and benefit as the vertical axis. This figure shows that despite the collective impact of various factors (types of contact networks, mean number of contacts per individuals and transmission probability), the magnitude of the benefits provided by contact tracing can be characterized in terms of the growth rates. For the combinations in which the growth rate is low, 1-hop contact tracing alone can sufficiently contain the virus, and 2-hop and 3-hop contact tracing do not provide notable further benefit in terms of reduction in the outbreak size. Specifically, when growth rate is less than or equal to 0.105, 1-hop contact tracing reduces the outbreak size by 85.0–99.9% (median 97.8%). Next, a sharp *phase transition* is exhibited once the growth rate crosses a threshold value, that is, the benefit that 1-hop provides dramatically decreases, and multi-hop contact tracing offers substantial further benefit. Specifically, when the growth rate exceeds 0.105, 1-hop contact tracing reduces the outbreak size by 5.6–74.5% (median 28.1%), while 2-hop and 3-hop contact tracing, respectively, reduce the outbreak size by 54.5–99.92% (median 97.6%) and 84.6–99.96% (median 99.8%). This suggests that, as the virus spreads faster, traditional contact tracing becomes less than adequate. This is because in the time that elapses between when an individual becomes infectious and *i* is quarantined through 1-hop contact tracing, the disease spreads from *i* through a chain of several hops, i.e. the contacts *i* infects infect their contacts and so on. In this case, as shown in figure 1*a*, pre-emptively tracing and quarantining multi-hop contacts can help tracing catch up with the speed of virus spread faster than 1-hop contact tracing.

**Figure 3.**
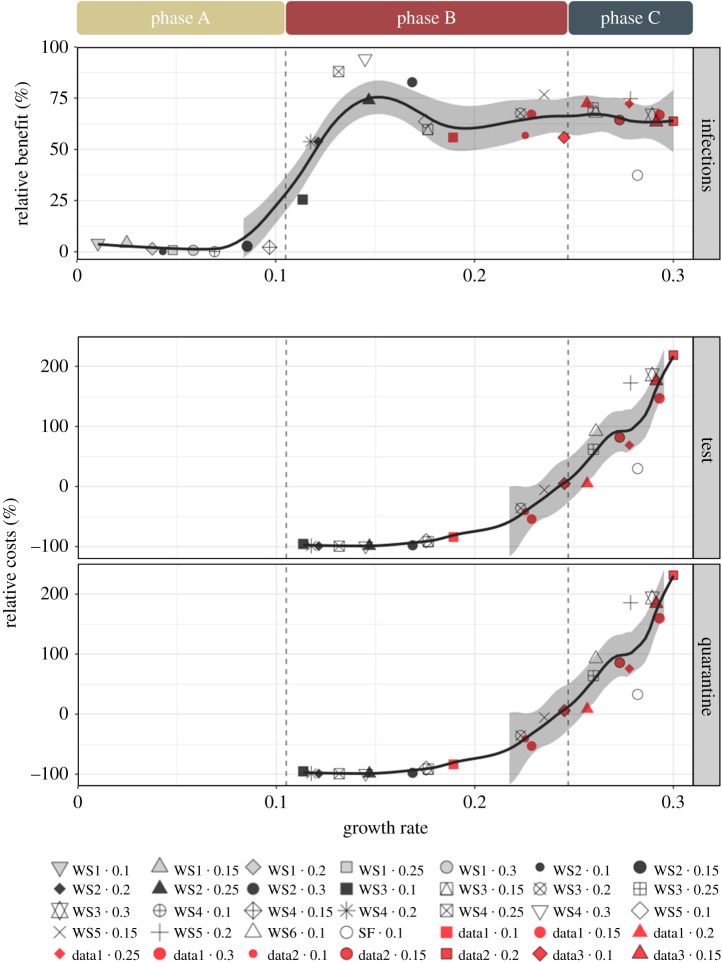
Relative benefit (the plot on top) and relative costs (the two plots on the bottom) are shown for 3-hop contact tracing under the default setting as a function of the growth rate. One can observe that the cost–benefit trade-offs can be classified into three phases, depending on the value of the growth rate. Each point represents a combination of contact patterns and transmission probabilities and corresponds to a growth rate on the *x*-axis. For example, WS2 *·* 0.25 represents that the contact pattern of a WS2 network (among the networks listed in table 2) and the transmission probability is 0.25, and corresponds to the growth rate value of 0.147. The relative benefit and relative costs for the data-driven networks (shown in red) behave similarly to those observed at the same growth rates in synthetic networks (shown in non-red). The solid lines correspond to the LOESS smoother with a span value of 0.3 and the shadings represent the 95% confidence interval around the smoother line. We determine the boundary between phases A and B as follows: compute the median value between the highest growth rate in phase A and the lowest growth rate in phase B for the synthetic networks and denote this value as the boundary. The boundary between phases B and C is defined similarly. The boundaries are depicted by lines.

We observe that 3-hop contact tracing can reduce the outbreak by 84.6–99.98% (median 99.8%) over the entire range of growth rates, as shown in figure 2. Hence, we quantify the costs and benefit of 3-hop contact tracing in comparison with 1-hop contact tracing. Figure 3 reveals that the relative benefit and relative cost for 3-hop contact tracing follow three *phases*:

— Phase A: In this phase, the relative benefit, as compared with single-hop contact tracing, is small (less than or equal to 20%). Here, this corresponds to the growth rates less than 0.105.
— Phase B: In this phase, the relative benefit increases substantially as compared with 1-hop while fewer total tests and fewer total sum of days of quarantine are needed for the entire population (relative benefit greater than 20% and relative costs less than or equal to 0%). Here, this corresponds to the growth rates between 0.105 and 0.247.
— Phase C: In this phase, multi-hop still provides a significant relative benefit, but requires greater costs compared with 1-hop tracing (relative benefit greater than 20% and relative costs greater than 0%). Here, this corresponds to the growth rates larger than 0.247.

Multi-hop contact tracing may incur higher costs than 1-hop contact tracing because it traces up to more hops even from the same number of confirmed cases. However, this can more rapidly mitigate the spread of virus when compared with 1-hop contact tracing through faster identification and quarantine of multi-hop contacts of infected individuals, thus fewer individuals need tests with passage of time. In phase B, the latter phenomenon dominates, in phase C the former.

#### 3.1.2. Data-driven networks

The above-mentioned simulation results on synthetic networks suggest that the cost–benefit trade-off can be classified into three phases, depending on the value of the growth rate. We now verify this phenomenon on data-driven network. Recall that the parameter *r* represents the percentage of contacts between individuals of different villages. For different values of the parameter *r*, the growth rates for the networks fall into the regimes of intermediate–high. The relative benefit and relative costs behave similarly to those observed at the same growth rates in synthetic networks (see, e.g. the red points that correspond to data-driven networks in figure 3). Thus, the trade-offs for the data-driven networks are consistent with those observed in the synthetic networks.

Next we study the role of different values of attributes, involving variations of false negative rates, false positive rates, turnaround times, starting times of contact tracing and cooperativity. To this end, we revert to our synthetic networks and classify phases A, B and C using the same criteria as in the default setting. For almost all variations of the attributes above (except for a debilitating form of non-cooperations that we will discuss), the cost–benefit trade-off for multi-hop contact tracing can be still classified into three phases, with sharp transitions between them, depending on the value of the growth rate. In this respect, the classification of the phases remains largely stable to variation of the above-mentioned attributes; the growth rate corresponding to the boundaries between phases tends to remain the same or shift to the left, as the environments become more challenging (electronic supplementary materials, figure S1). The only exception is when we consider variation of the most debilitating form of non-cooperations (non-cooperative individuals not revealing their contacts, not testing or quarantining). In this case, the cost–benefit trade-off is significantly altered because refusal to test and reveal contacts limit tracing and the contact network as known to tracers becomes highly fragmented and sparse. See the electronic supplementary materials for more details and results on the impact of different values of attributes.

### 3.2. Comparison of contact tracing to complete lockdown

We compare the multi-hop contact tracing strategy with a total quarantine approach (complete lockdown). Lockdowns have been implemented in many parts of the world, with different lengths of lockdowns and different degrees of rigor from region to region. For example, Wuhan, China, has been the first in the world to implement the complete lockdown for 76 days, starting January 2020. Subsequently, Italy and Spain, respectively, imposed the first and the second total lockdown in Europe and lasted 56 days and 66 days, starting March 2020 [19]. Furthermore, there are many countries which imposed lockdown with different stringency levels for more than 100 days (e.g. in Argentina, Azerbaijan, Bolivia, Nepal, UK, Peru, Saudi Arabia, Czech Republic, Greece, Germany, Ireland and Australia) [20].

We consider the quarantine cost for lockdown as the number of person-days of stay-at-home inflicted due to lockdown. This can be considered as the product of the total population size and the duration of the lockdown. This assumes complete compliance by the populace, which has not been realized in many places where such an order was issued. Note that it becomes difficult to assess the cost if compliance is less than perfect, as the degree of compliance is rarely ever reported accurately. This is why we consider the case of Wuhan, where by all accounts the compliance was near total. Assuming complete compliance, we consider the best case scenario for complete lockdown—that the total number of infections during the lockdown is 0.

We compare the quarantine cost for lockdown with that for contact tracing during the respective periods in which the two are implemented. Recall that contact tracing is implemented for six months, which is longer than the lockdown period we consider (76 days for Wuhan). We do not compare the number of tests because different places implemented different testing strategies, and in principle if lockdown can be completely enacted, every individual will be isolated from every one else (which is of course impractical) and one need not even test anyone. We also compare the fraction of populace infected during the respective periods of consideration for contact tracing and complete lockdown (the fraction is assumed to be 0 for the latter).

**Figure 4.**
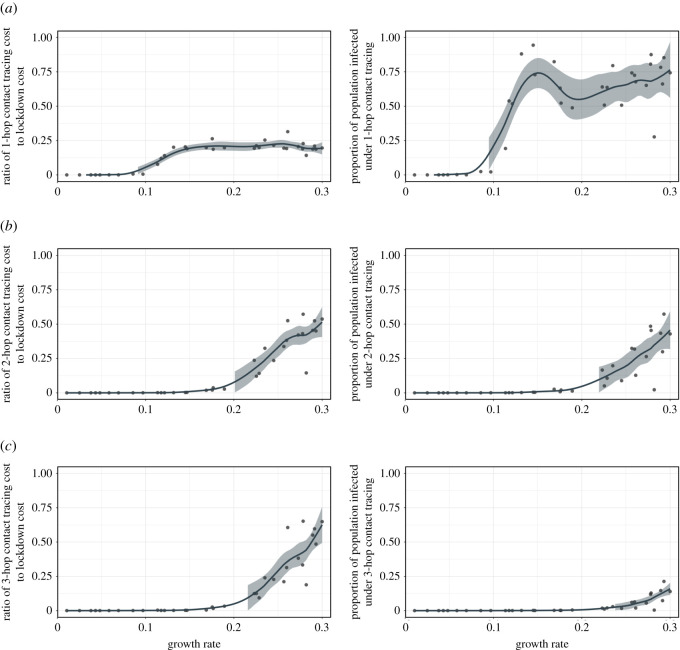
(*a*) (left) The ratio of quarantine cost for 1-hop contact tracing to quarantine cost for lockdown as a function of the growth rate; (right) the proportion of population infected under 1-hop contact tracing as a function of the growth rate. (*b*) Same figures as (*a*), but under 2-hop contact tracing instead of 1-hop. (*c*) Same figures as (*a*), but under 3-hop contact tracing instead of 1-hop. For all figures (*a*–*c*), each point represents a combination of contact patterns and transmission probabilities and corresponds to a growth rate on the *x*-axis. We use the same contact patterns and transmission probabilities considered in figure 3, but omit the labels for points. The solid lines correspond to the LOESS smoother with a span value of 0.3 and the shadings represent the 95% confidence interval around the smoother line.

The quarantine cost for 1-hop contact tracing is lower than 1/4 of that for lockdown for the entire spectrum of growth rates we consider (left panel of figure 4*a*). But except for growth rates less than 0.1, the total number of infections is quite high for 1-hop contact tracing (the right figure of figure 4*a*).

On the other hand, multi-hop contact tracing behaves differently. Multi-hop contact tracing sufficiently contains the virus with even lower cost compared with lockdown. As shown in the right figures of figure 4*b*,*c*, the fraction of populace infected for (i) 2-hop contact tracing is near 0 for most of the growth rates, e.g. up to 0.2, and (ii) 3-hop is close to zero in all cases except a few in higher range of growth rate. Nonetheless, the quarantine cost for both 2-hop and 3-hop are far lower than that for complete lockdown in all cases; the ratio with that for complete lockdown is near 0 for most of the growth rates (up to 0.2) (the left figures of figure 4*b*,*c*). Thus multi-hop contact tracing policy has a comparative advantage over lockdown in most cases. Summarily, overall, multi-hop contact tracing can effectively control the spread of the virus while eliminating a large volume of unessential quarantines through selective testing and quarantining.

**Figure 5.**
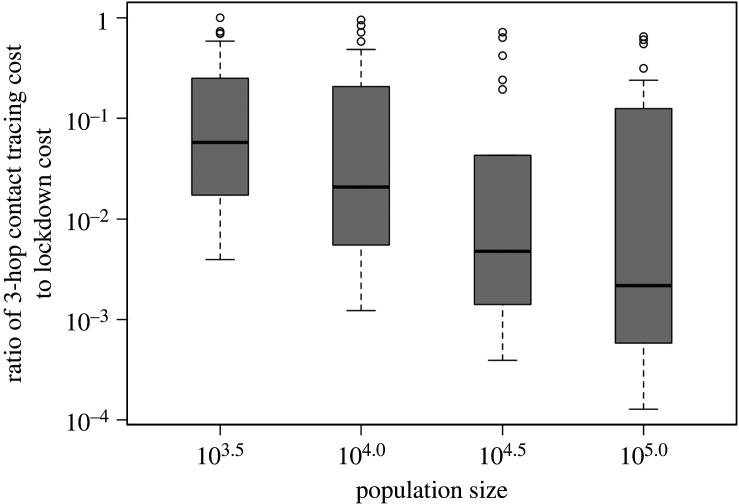
Box plots showing the ratio of quarantine cost for 3-hop contact tracing to quarantine cost for lockdown as a function of population size, across combinations of various synthetic networks and transmission probabilities used in figure 4. The boxes represent the 25th (Q1), 50th (median) and 75th (Q3) percentiles; the whiskers represent Q1 − 1.5 × IQR and Q3 + 1.5 × IQR, where IQR (interquartile range) is the difference between Q3 and Q1. The median tends to decrease as population size increases. The *y*-axis is in logarithmic scale.

We now compare the quarantine-cost advantage of multi-hop contact tracing over lockdown as a function of the population size. Figure 5 represents the ratio of quarantine cost for 3-hop contact tracing to quarantine cost for lockdown as a function of population size, across combinations of synthetic contact patterns and transmission probabilities we considered in figure 4. This figure shows that the ratio tends to decrease as population size increases. Thus, the more populated the region, the more pronounced the quarantine cost advantage of multi-hop contact tracing over lockdown. This is an important consideration because in real life sizes of population subjected to lockdown are really large (e.g. 8.5 million people live in Wuhan).

Finally, as noted before, the number of tests needed in lockdown can be low; may even be 0 when there is perfect compliance. While this may come across as a significant advantage of lockdown, there are several other immeasurables of strong adverse impact associated with the quarantine cost of lockdown. Prolonged quarantines lead to substantial increase in non-COVID-19 deaths [21]. For example, in the USA, it is estimated that an additional 250 000 people with preventable cancer died annually due to delays in screening and treatment [22]. These delays are exacerbated due to person-hours lost in quarantines. In Ontario and British Columbia, Canada, deaths from drug overdose have seen a surge since the lockdown began, potentially due to isolation [23]. Domestic violence has increased during lockdowns (e.g. 30% increase in France, 25% in Argentina) [24]. Economies have been devastated globally during lockdowns, particularly in the poor countries [25,26]. Also, among other things, a study [27] that performed a meta-analysis found that, despite inflicting exorbitant economic and social costs, the lockdowns in spring 2020 had little or no impact on COVID-19 mortality rates, possibly because longer lockdown periods ensure lower compliance due to increased fatigue [28]. The adverse impact due to all the above are difficult to quantify. Nonetheless, all in all, significantly higher quarantine cost of lockdown may accordingly outweigh other considerations.

### 3.3. Diminishing returns for increasing number of hops

We have shown that the cost–benefit trade-offs can be classified into three phases, with transitions between them, depending on the value of the growth rate, and can be substantially enhanced through the deployment of a natural multi-hop generalization through comparison between traditional (1-hop) and 3-hop contact tracing. In this section, we investigate the highest number of hops that can lead to a non-negligible further reduction in the number of infections. Our numerical computations in this section reveal that most of the substantial benefit that multi-hop contact tracing provides can usually be attained within 3-hops, and considering 4 or a larger number of hops is usually redundant as the further benefit of adding more hops is negligible. This is because the further benefit for adding another hop beyond 1-hop tends to diminish progressively, thus a saturation phenomenon in which the benefit increases only marginally by increasing the number of hops beyond a certain point arises. Hence, increasing the number of hops beyond the hop number at which the saturation phenomenon is observed is not effective when it comes to reducing the number of infections. And the saturation phenomenon is usually observed within 3 hops. Our criteria is that the saturation point becomes *k*-hop when further benefit provided by (*k* + 1)-hop over the previous *k*-hop is less than 10% (i.e. difference between benefits of (*k* + 1)-hop and *k*-hop is less than 10%).

**Figure 6.**
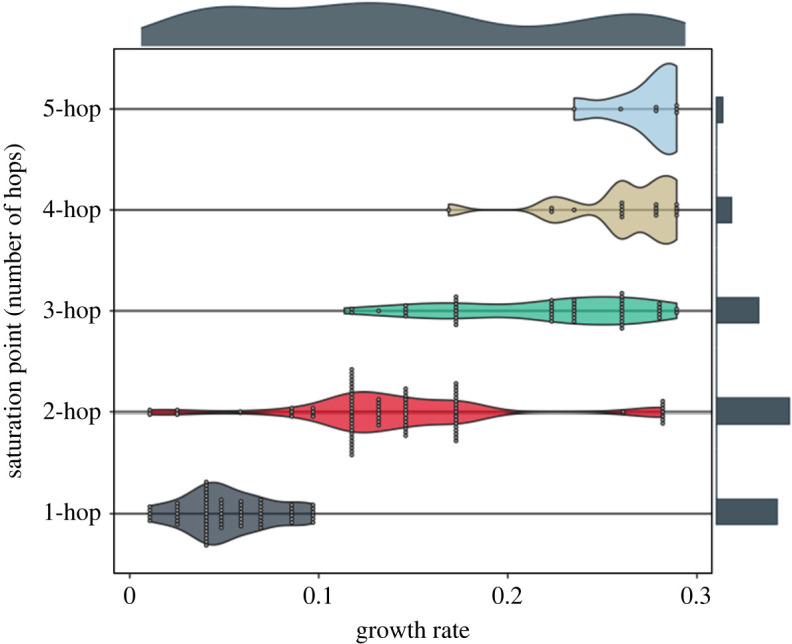
Violin plots show the distribution of growth rate across discrete saturation points, based on the pooled data across the environments (involving variations of false negative rates, false positive rates, turnaround times, starting times of contact tracing and cooperativity (Scenario 1)). We use the same synthetic networks and values of transmission probability that are used in figure 3. Each point corresponds to a certain contact pattern, a certain transmission probability and a certain environment, which determine the growth rate (*x*-axis). Marginal distributions are added to the margin of each axis. The saturation point becomes greater as the growth rate increases; the instances of the saturation point being 1 hop is mostly concentrated in low growth rate region, 2 hops in intermediate region, and 3 hops and more in high region. Furthermore, marginal histogram along the *y*-axis shows that the saturation point is confined to 1, 2 and 3 hops in 89% of all instances, while the saturation point becomes 4 or 5 hops in 11% of instances corresponding to higher growth rates in more challenging environments.

We pooled all the results across variations of practical aspects of contact tracing and testing (involving variations of false negative rates, false positive rates, turnaround times, starting times of contact tracing and cooperativity (Scenario 1)), and observed that there are broad trends, with regard to the saturation points, across variations of the environments above. Specifically, the saturation point becomes greater as the growth rate increases (figure 6) or the environments become less conducive toward containing the disease. Even for wide variations of the attributes above, marginal histogram along the *y*-axis in figure 6 shows that the saturation point is confined to 1, 2 and 3 hops in most cases (89% of all instances), while the saturation points of 4 and 5 hops largely arise for very limited conditions such as higher growth rates in more challenging environments (11% of all instances).

In addition to the saturation points, we seek to reveal some broad trends with regard to the choice of the number of hops, recurring across various environments, taking into account both further benefit and cost incurred by each hop over the previous. Our simulations show that the broad trends resemble the trends with regard to the saturation points; as growth rate increases or environments becomes more challenging, the cost–benefit trade-offs propel us towards choosing higher number of hops. Refer to electronic supplementary materials for details on specific numbers, analysis, and criteria for choosing the number of hops, in which we present the further benefits and costs up to 5 hops for different parameter combinations.

## 4. Discussion

Contact tracing has been deployed during the first year of the pandemic in many countries, but very few of those countries have successfully contained the pandemic before the advent of pharmaceutical interventions. Vietnam is one of the few success stories in successful containment of the outbreak in early stage of pandemic, and it is also the only country to have incorporated multi-hop contact tracing in its containment programme. Since contact tracing (and quarantine) remains one of the few available mechanisms to contain the outbreak and prevent a pandemic during the early phases of any epidemic, an independent influence of the multi-hop contact tracing policies in the absence of any public health intervention needs to be comprehensively and systemically investigated. It is crucial to examine when and why traditional contact tracing is not sufficient to contain the virus and whether the multi-hop contact tracing can enhance the cost–benefit trade-offs in such circumstances.

In this work, we embarked on an investigation of multi-hop contact tracing considering a diverse set of large-scale contact networks, spanning synthetic networks of various families and design choices and those obtained from real-world interaction data. We now summarize and position our results. First, our findings confirm the intuition that multi-hop contact tracing reduces the spread of the infection. Next, our findings, however, go beyond, by revealing patterns that cannot be intuited *a priori*. We reveal that multi-hop contact tracing has the potential to reduce the outbreak to a much smaller size when compared with conventional contact tracing (i.e. 1-hop contact tracing), *even at lower costs* than the conventional contact tracing. We also show that the cost–benefit trade-offs for multi-hop contact tracing can be classified into three phases, with sharp transitions between the phases, and each phase corresponds to a different range of the initial epidemic growth rates. When the growth rates are low, multi-hop becomes redundant as single-hop contains the outbreak adequately. For higher growth rates, multi-hop substantially reduces the outbreak size, incurring (i) substantially lower quarantining and testing costs as compared with single-hop in the intermediate growth rate region and (ii) considerably higher costs in the high growth rate region. Furthermore, the classifications of the phases turn out to be robust to wide variations of almost all the practical aspects of contact tracing and testing.

Third, we show that the further benefit of adding another hop beyond 1-hop tends to diminish progressively, thus saturation phenomenon arises. While it is intuitive that there would be a saturation phenomenon, the impact of the growth rates on the saturation point in different ambiences cannot be inferred without the quantitative investigation. As growth rate increases or the contact tracing and testing ambience becomes more challenging, the saturation point becomes greater and the cost–benefit trade-offs propel us towards choosing higher number of hops. We calculate the growth rates in a large number of political units from publicly available pandemic data; our calculations show that these growth rates span all three ranges. In particular, therefore, multi-hop contact tracing substantially reduces the outbreak size and lowers overall costs for a large number of realistic values of growth rates.

Multi-hop contact tracing has been subject to limited rigorous investigation thus far. To our knowledge, the only other work to investigate this concept has been [29]. Our work is complementary to [29] which used real-world social network data of 468 individuals and considered tracing and quarantining (without testing) both primary and secondary contacts of those who test positive. Firth *et al.* [29] found that quarantining secondary contacts decreases the cumulative infection count compared with quarantining only the primary contacts, but also requires substantially higher number of quarantines. Next, they focused on reducing the number of quarantines through (i) social distancing and (ii) testing. When individuals are tested, those who test negative are released from quarantine right after the results are obtained; this reduces the quarantine periods but increases the outbreak. The authors acknowledge that it is unclear if their results would hold for networks with larger size. Results may become artefacts of network size for multi-hop contact tracing because the length of contact chains may be limited by network size when the size is small. We investigate multi-hop contact tracing involving a combination of quarantining and testing for *k* hops, where *k* can be 2, 3, 4, 5, etc., over large networks comprising up to 100 000 individuals, and consider a large number of instances from both synthetic networks corresponding to various families and parameter choices and networks obtained from contact data. We use tests to further trace contacts rather than to release those traced early from quarantining and evaluate both testing and quarantining costs. As mentioned in the previous paragraph, we show that the cost–benefit trade-offs for different number of hops (1, 2, 3, 4, 5, etc.) can be very different depending on growth rate and venue of the tests, and the trade-offs for different test venues can be characterized by only one parameter vis-à-vis the network topology, that is the growth rate. In particular the result [29] reports as to the comparison between 1 and 2 hops for ‘quarantine only’ corresponds to what we observe throughout the high growth rate range for our simulations. When social distancing is additionally incorporated, the growth rate decreases; their finding in this case is consistent with the phenomenon we observe for the intermediate growth rate range. Thus, our investigation positions their findings as parts of a broader trend. This is in addition to revealing the phase-transition patterns for cost–benefit trade-offs and identifying the hop choices for different ranges of growth rates, different testing ambiences and a diverse class of larger networks.

We now discuss limitations of multi-hop contact tracing in the current context and how to circumvent the limitations in order to prevent a future epidemic from becoming a pandemic. First, the benefits of contact tracing, both single hop and multi-hop, considerably decrease if a non-negligible percentage of the society do not reveal their contacts, do not test, and do not quarantine when asked to. Cooperation with health authorities varies across the world: while a high degree of cooperation was witnessed in South Korea and Taiwan which had suffered from large-scale epidemics in the last 20 years [30,31], cooperation was lower in Europe and the USA [32], both of which experienced a large-scale epidemic about a century ago (the 1918 flu). Learning from the experience of this pandemic, public awareness campaigns need to be pursued to elicit cooperation with health authorities. Multi-hop may provide an important advantage to ensure cooperation in that it can contain the outbreak faster which may incentivize full cooperation for a short duration, whereas cooperation may wane due to pandemic fatigue as time progresses. Furthermore, there are challenges that the real-world implementation of multi-hop contract tracing may face. These challenges can, however, be overcome by digital contact tracing apps, albeit various challenges and concerns need to be addressed. For example, in democracies the digitization is often critically reliant on the willingness of the populace to download the apps the health authorities use, which has again varied from country to country for the COVID-19 outbreak. For example, in Singapore over 92% of the population over 6 years of age had downloaded the governmental contact tracing app on their smartphone [33], but the fraction has been lower in many other countries, particularly those in Europe and the USA.

We next describe the generalization of our framework for an investigation for other infectious diseases. Each infectious disease differs from the other in two aspects such as stages of the disease evolution and parameters for the disease. The investigation on cost–benefit trade-off of multi-hop contact tracing for an arbitrary infectious disease can be extended by appropriately choosing the stages and parameters for the disease based on our framework. Almost all infectious diseases include susceptible, recovered and dead stages, the choice of other states allow us to cater for a specific disease in question. As for COVID-19, latent, pre-symptomatic, symptomatic and asymptomatic are the additional stages. Let us consider smallpox as an example of another infectious disease. All individuals infected with smallpox develop symptoms (fever and rash), thus latent and symptomatic stages can be added and the symptomatic stage can be further subdivided into fever, early rash and late rash stages. Smallpox does not have asymptomatic carriers, so the asymptomatic stage can be omitted [34]. The different stages and parameters for the disease in question alter the epidemic growth rate. The question that remains is that if the observed patterns regarding the cost–benefit trade-off for multi-hop contact tracing, namely the phase classifications, the sharp phase transitions and the saturation phenomenon, extends to other infectious diseases. Investigating multi-hop contact tracing for other infectious diseases based on our framework constitutes an imperative direction for future research towards building a knowledge-base for containing future epidemics before they become pandemics and repeat the enormous toll that COVID-19 imposed.

We now describe topics for future research. Stochastic simulations are significantly more computationally intensive than analytical approximation-based evaluation. Determining analytical approximations for accurately evaluating testing strategies in complex heterogeneous networks therefore constitutes an important direction for future research. It may be possible to further adapt and refine the analytical approximation strategies already developed (e.g. [13–16]) for evaluating other control strategies for infectious diseases for example. Next, in this paper, we have assessed the total costs and benefits to the overall populace for an important class of testing strategies; for this purpose, we have considered the fraction of individuals who cooperate with the testing requirements as a parameter. In practice, whether to cooperate with testing requirements is an individual behavioural choice which every individual arrives at depending on his perception of his individual costs and benefits from such compliance; the individual choices also evolve over time depending on how individuals perceive the choices of their peers and their observations of their ambiences. An interesting direction for future research would be to devise a framework to determine the equilibrium value of the overall fraction of compliant individuals as individual choices evolve. Such frameworks have already been developed for example for other behavioural choices pertinent to the control of infectious diseases, e.g. whether to vaccinate [11], whether to wear masks [12], etc., drawing from tools such as evolutionary game theory for example; these therefore suggest possible directions for approach for modelling behavioural choices pertinent to testing.

We have assumed that the traced *k*-hop contacts of an individual (say *i*) who tests positive can start their quarantine within a day of *i* testing positive, though they test after some delay. But this is not in general possible unless *i* downloads the contact tracing app either before or at least right after testing positive [35]. Next, depending on classifiers such as duration, environment (indoor or outdoor), usage of protective equipment, observance of personal hygiene, different contacts may pass on infection with different probabilities. Assuming that such a probability is identical for all contacts with same infectious categories, which is what we did, is equivalent to considering an average over all contacts. Explicitly investigating the impact of (i) delays in starting quarantining and (ii) non-uniform transmission probabilities also constitute directions for future research.

## 5. Methods

### 5.1. Stochastic simulations of virus transmission

**Figure 7.**
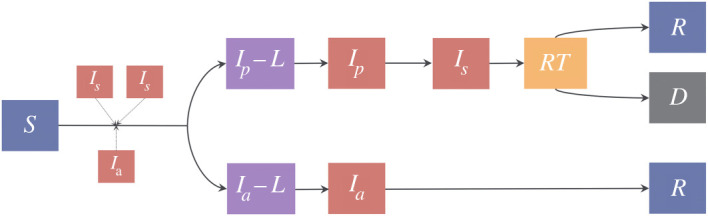
Virus transmission model illustration. The compartmental model consists of the following compartments: susceptible (*S*), presymptomatic-latent (*I*_*p*_ − *L*), presymptomatic (*I*_*p*_), symptomatic (*I*_*s*_), ready-to-test (*RT*), asymptomatic-latent (*I*_*a*_ − *L*), asymptomatic (*I*_*a*_), recovered (*R*) and dead (*D*).

Compartmental models have been widely used in studies on virus spread [9,10]. We use a discrete time compartmental disease model to simulate the progression of COVID-19 where the transition from each compartment to the next happens after a random amount of time with a geometric distribution. The disease propagation is mechanistically simulated on a given network (see table 2 for various networks we consider). Different stages of the disease are shown in figure 7. The model consists of the following stages: susceptible (*S*), presymptomatic-latent (*I*_*p*_ − *L*), presymptomatic (*I*_*p*_), symptomatic (*I*_*s*_), ready-to-test (*RT*), asymptomatic-latent (*I*_*a*_ − *L*), asymptomatic (*I*_*a*_), recovered (*R*) and dead (*D*). Only symptomatic individuals show symptoms, while presymptomatic, symptomatic and asymptomatic individuals can infect others. When a susceptible individual comes into contact with an infectious individual, the susceptible is infected with transmission probability *β*.

Once an individual is infected he becomes contagious after a geometrically distributed latency time, the expectation of which depends on whether he will develop symptoms at some point or otherwise. Following the nomenclature in compartmental models already used for COVID-19, we assume that an infected individual becomes asymptomatic-latent (with probability *p*_*a*_) or presymptomatic-latent (with probability 1 − *p*_*a*_) and those in this latency period have a negative test (the tests do not detect the presence of COVID-19). The asymptomatic-latent (*I*_*a*_–*L*) individuals never develop symptoms, do not infect others for a mean latency duration of 1/*λ*, and subsequently become contagious, at which stage we call them asymptomatic or *I*_*a*_ for simplicity. An asymptomatic individual remains contagious for a geometrically distributed random duration with mean 1/*r*_*a*_, after which the individual recovers. We now consider the other compartment an individual enters after infection, the presymptomatic-latent compartment. A presymptomatic-latent individual, say *B*, becomes contagious after a mean latency period of 1/*λ*, at which point we call *B* presymptomatic or *I*_*p*_. *B* remains presymptomatic for a geometrically distributed duration with mean 1/*α*; after this duration *B* develops symptoms and is called symptomatic. A symptomatic individual *B* continues to infect contacts until *B* opts for testing (*RT*). The duration for which a symptomatic individual infects others is geometrically distributed with mean 1/*w*. Once this duration ends, the patient quarantines and does not infect others. The patient ultimately dies (*D*) with probability *p*_*d*_, or recovers (*R*) with probability 1 − *p*_*d*_, after a geometrically distributed duration with a mean of 1/*r*_*s*_. We do not consider that individuals can be reinfected. In all the networks, we consider that initially all but three individuals are susceptible, among the three there is one presymptomatic, one symptomatic and one asymptomatic. We eliminate all interactions involving individuals in quarantine (either due to contact tracing or developing symptoms). Refer to table 1 for the parameter values we choose.

**Table 1.** Values of disease parameters.

parameter	notation	value	reference and description
transmission probability	*β*	[0.1, 0.3]	assumed various scenarios considering [36,37]
proportion of infections that are asymptomatic	*p*_*a*_	0.4	[38,39]
mean latency period	1/*λ*	2 days	Inferred from [40]
mean duration in asymptomatic stage	1/*r*_*a*_	7 days	Inferred from [40,41]
mean incubation period (period between infection and symptom onset)	1/*λ* + 1/*α*	5 days	[42,43]
mean duration from symptom onset to testing	1/*w*	4 days	inferred from [44]
mean duration of symptom onset to recovery/death	1/*w* + 1/*r*_*s*_	14 days	inferred from [38,41]
fraction of symptomatics who die	*p*_*d*_	0.0065	[38]

### 5.2. Initial epidemic growth rate

We define the initial epidemic growth rate in the target region as

5.1 $$\mathrm{Growth}\;\mathrm{Rate}=\frac{\ln N_{t}-\ln N_{0}}{t-t_{0}},$$

where *N*_*t*_ and *N*_0_, respectively, are the cumulative number of infected individuals on day *t* and day *t*_0_ in a target region (i.e. growth rate during the period [*t*_0_, *t*]). The growth rate is in units of day^−1^. We consider that local community transmissions begin at *t*_0_, where start date *t*_0_ is the time at which 40 cases are recorded in the unit and end date *t* is *t*_0_ + 21 (three weeks from *t*_0_). This attribute depends on the network structure and the transmission probability *β*. We choose this expression (particularly the logarithmic functions) because the growth of infections during the initial period has been widely observed to be exponential for different epidemics including the COVID-19 pandemic. We consider an initial period because the growth of the epidemic in this period typically happens before any public health intervention, such as contact tracing, pre-emptive quarantining, lockdown, etc., and therefore represents the innate speed of the spread of the virus in the network, and depends only on the network structure and *β*.

Using the data available in [18], we calculated this quantity for COVID-19 for different political units (country/region or province/state/dependency). We limited the analysis to political units that recorded at least 40 cases within the early stages of spread of COVID-19 (i.e. within first three months up to 20 April 2020) of the pandemic. There are 142 such countries/regions and 116 province/state/dependencies. We found that the growth rates in all these political units range from 0 to 0.31, with a median of 0.12 (electronic supplementary materials, table S1).

### 5.3. Practical aspects of contact tracing and testing

We consider various attributes that affect the efficacy of contact tracing, involving variations of false negative rates, false positive rates, test result turnaround times, starting times of contact tracing and level of cooperation with contact tracing and testing. We first set a default scenario and then consider a variety of environments departing from the choices in the default scenario based on estimates available in the literature. We first consider attributes of the testing equipment and logistics. Test results may be inaccurate, suffering from *false-negatives* and *false-positives*. A review [45] of 34 studies based on 12 057 confirmed patients showed that false-negative rates ranged from 1.8 to 58%, with a median of 11%. We thus set the median 11% false-negative rate as default, but consider both the lowest and highest end-points of the reported range, though note that 58% is unrealistically high for the test-result to be meaningful. As for false positives, studies assessing a total of 119 South Korean laboratories [46,47] and 52 Austrian laboratories [48] did not report false positive results, and a study evaluating 365 laboratories in 36 countries reported a false positive rate of 0.7% [49]. We set the 0% false-positive rate as default, but also consider 0.7% rate. Next, note that there is usually a delay between when a test is conducted and its result is obtained, this delay is known as the turnaround time. According to CDC [50], the turnaround times for (i) most nucleic acid amplification tests (NAATs), such as RT-PCR, vary between 1 and 3 days, and (ii) point-of-care tests are 15–45 min. We set default value of the turnaround time as 1 day, but also consider 3 days.

Public health authorities in different political units may decide to start contact tracing when the infection level in the target populace crosses a certain threshold. We consider that contact tracing is initiated when the first individual tests positive as the default option. This is in accordance with the observations of the leading practitioners of contact tracing programmes who recognize that contact tracing should start as soon as the first case is diagnosed. Once the outbreak spreads, the logistical challenges associated with contact tracing multiply because of the sheer volume of the contacts that need to be traced [51]. Also, the only countries to have successfully contained the outbreak through contact tracing (i.e. before pharmaceutical preventives became available), namely South Korea, Japan and Vietnam, started the process early [6]. In order to understand the impact of the delayed initiations, we also consider the cases, e.g. six months and a year from when the outbreak is recorded. Using the data available in [18,52], we calculated the percentage of cumulative confirmed cases in different political units (186 countries and 137 states/provinces/dependencies) at the end of six months and a year from the date the datasets were recorded. The median of the percentages is 0.1% for six months delay and 1.1% for a year delay. Accordingly, we also consider scenarios in which contact tracing is initiated when the percentage of cumulative infections reaches 0.1% and 1.1%.

Finally, we assume full cooperation from the target populace as the default setting, i.e. every individual tests and quarantines as instructed by his local public health authority and reveals his contacts to them. But, we also consider scenarios in which cooperativity is less universal.

## 6. Data

In the contact networks, the nodes represent the individuals and the edges their contacts; the degrees of the nodes represent the number of contacts of the corresponding individuals. Growth of an epidemic depends on structural attributes of the contact networks, such as (i) average path lengths between nodes, (ii) clustering coefficient, and (iii) degree distribution. We consider two broad classes of synthetic networks, which captures different ranges of the above attributes: (i) Watts–Strogatz networks [53], and (ii) scale-free networks [54]. Additionally, we consider a social contact network obtained from data recorded from social and professional interaction patterns that have been realized in practice.

By considering all of these diverse networks complementing each other in fundamental characteristics, we are able to assess the cost–benefit trade-offs of multi-hop contact tracing and testing strategies for widely varying contact patterns. See table 2 and below for details on all the synthetic and data-driven networks, we consider.

**Table 2.** Contact networks.

notation	types of networks	population *N*	average degree 〈*k*〉	diameter *d*	average path length *l*	average clustering coefficient 〈*C*〉
WS1	Watts–Strogatz network w/ *p* = 0.01	100 000	4.00	95	39.3	0.472
WS2	Watts–Strogatz network w/ *p* = 0.1	100 000	4.00	22	12.4	0.275
WS3	Watts–Strogatz network w/ *p* = 1	100 000	4.00	17	8.42	0.0000413
WS4	Watts–Strogatz network w/ *p* = 0.01	100 000	8.00	32	16.6	0.606
WS5	Watts–Strogatz network w/ *p* = 0.1	100 000	8.00	11	7.50	0.349
WS6	Watts–Strogatz network w/ *p* = 1	100, 000	8.00	10	5.77	0.0000804
SF	scale-free network	100 000	4.00	10	5.88	0.000651
DATA1	data-driven network w/ *r* = 1	69 441	8.49	17	8.81	0.627
DATA2	data-driven network w/ *r* = 3	69 441	8.49	15	7.45	0.589
DATA3	data-driven network w/ *r* = 5	69 441	8.49	15	6.97	0.553

*Notes.* *p* is the rewiring probability of Watts–Strogatz networks, and *r* is the mixing parameter in data-driven network. The average degree is the average number of edges per node. The distance between a pair of nodes is the length of the shortest path between them. The diameter is the maximum value of this distance over all pairs of nodes. The average path length is the average of this distance over all pairs of nodes; only the lengths of the existing paths are considered and averaged. Clustering coefficient of a node *i*, *C*_*i*_, is defined as the ratio between the actual number of links between the neighbours of *i* and the maximum possible number of links between the neighbours of *i* [53]. This is high if there exists a large number of edges in the neighbourhood of *i*. The average clustering coefficient, 〈*C*〉, is the average of *C*_*i*_ over all nodes *i*. The average degree, average path length and average clustering coefficient are rounded to three significant figures.

### 6.1. Synthetic networks

#### 6.1.1. Watts–Strogatz networks

Each network we consider has *N* = 100 000 nodes. The Watts–Strogatz networks have average degrees of 〈*k*〉 = 4, 8, that is, 200 000 and 400 000 edges. They are generated following a variant of the original Watts–Strogatz model. Based on a ring of *N* nodes, each node is connected to *k* nearest neighbours by undirected edges. Subsequently, each endpoint of each edge is rewired to a uniformly randomly chosen node over the entire ring with rewiring probability of *p*, avoiding link duplication (i.e. multiple edges between the same pair of nodes) and self-loops. By varying a parameter, referred to as the *rewiring probability*, of the Watts–Strogatz networks from 0 to 1, one can realize (i) average path lengths that range from linear to logarithmic functions of the number of nodes, and (ii) clustering coefficients from high to vanishingly small [53]. Studies based on real data suggest that contact networks among individuals exhibit short (i.e. logarithmic) average path length and high clustering coefficients (commonly referred to as the small-world property) [55,56]. When clustering coefficient is high, most of the contacts happen between individuals in given phases or clusters; when clustering coefficient is low, most contacts happen between randomly selected individuals. Both extremes and values in between can be captured by choosing the value of the rewiring probability [53]. The special case of the Watts–Strogatz model in which the average path length is logarithmic and the clustering coefficient is low corresponds to a variant of the Erdős–Rényi random networks; we consider this variant as well.

#### 6.1.2. Scale-free networks

Each network we consider has *N* = 100 000 nodes. The scale-free network topologies are generated by the Barabási–Albert model where new nodes are added at each time step with *m* links that connect to existing nodes with a probability that is proportional to the degree of the existing nodes [54]; we set *m* = 2 to generate the network. The resulting network consists of 199 997 edges, thus average degree of a node is 〈*k*〉 = 3.99994. Scale-free networks exhibit heterogeneous degree distributions, i.e. the degree distribution has a high variance and only a polynomially decaying tail (‘fat-tailed’ distribution). Unlike scale-free networks, the degree distribution in Watts–Strogatz models have exponentially decaying tails for usual choices of parameters. The implication of this difference is that scale-free networks invariably have some individuals with very high degree, while the probability of the same happening in Watts–Strogatz models is low.

### 6.2. Data-driven network

We use the publicly available network data covering a wide range of interactions among individuals collected by survey in each of 75 villages located in Karnataka, India [57]. The surveys include interaction information such as names of those who visit the respondents’ homes, those with whom the respondents go to pray, etc. In this dataset, each village consists of 354–1775 individuals. The limitation of this dataset is that it contains information only on social interactions between individuals within each village. However, in reality, individuals living in different villages do come in contact, and pandemic spreads from one village to another through these contacts. Also, the cost–benefit trade-off for multi-hop contact tracing is best evaluated on large population sizes, otherwise the length of the contact chains will be limited by diameter of the contact network. We therefore introduce interaction between individuals in different villages through degree-preserving rewiring [58,59]. We first randomly select two villages and select a random edge within each cluster, and then swap the two edges to reach across the pair of villages. The process is repeated until the percentage of edges that are rewired among the total number of edges becomes *r*%, and *r* is called the mixing parameter [59]. The degree-preserving rewiring preserves the degree of all the nodes in the network regardless of the parameter *r*, but it changes the frequency of inter-village interactions and network properties. The resulting network consists of a total of 69 441 individuals and 294 945 interactions among them. We generated three different networks with the mixing parameters *r* = 1, 3, 5. As the parameter *r* increases from 1 to 5, the diameter, average path length and clustering coefficient monotonically decrease (refer to table 2).

## Data Availability

Data are included in this article and the electronic supplementary material [60]. Contact networks generated during this study and custom code are available from the repository https://github.com/jungyeol-kim/RSOS-contact-tracing, also available in Zenodo: doi:10.5281/zenodo.7106060 [61].
